# Prevalence and predisposing factors of depressive symptoms in patients with stable coronary artery disease: a cross-sectional single-center study

**DOI:** 10.18632/aging.102026

**Published:** 2019-06-17

**Authors:** Yeshun Wu, Bin Zhu, Zijun Chen, Jiahao Duan, Ailin Luo, Ling Yang, Chun Yang

**Affiliations:** 1 Department of Cardiology, The Third Affiliated Hospital of Soochow University, Changzhou 213003, China; 2 Department of Critical Care Medicine, The Third Affiliated Hospital of Soochow University, Changzhou 213003, China; 3 Department of Anesthesiology, Tongji Hospital, Tongji Medical College, Huazhong University of Science and Technology, Wuhan 430030, China

**Keywords:** depressive symptoms, stable coronary artery disease, elderly, lipoprotein, creatinine

## Abstract

The incidence of depressive symptoms in patients with stable coronary artery disease (SCAD) has significantly increased. However, its pathogenesis and treatment mechanisms are still incompletely understood. In this study, 144 patients with SCAD were recruited. Depressive symptoms of patients with SCAD were evaluated using Zung Self-Rating Depression Scale during hospitalization, and the patients were categorized into two subgroups: the non-depressive and depressive groups (further divided into mild and moderate/severe depressive groups). The rate of moderate/severe depressive symptoms in patients with SCAD was 18.8%. The mean age of patients in the depressive and mild depressive groups was older than that of those in the non-depressive group, and patients in the moderate/severe depressive group had higher high-density lipoprotein (HDL) and lower creatinine (Cr) levels. Binary logistic regression analysis showed that lower low-density lipoprotein (LDL) levels were significantly associated with increased risks of mild depressive symptoms, whereas higher HDL and lower Cr levels were significantly associated with moderate/severe depressive symptoms, suggesting that patients with SCAD were prone to experience depressive symptoms, especially in the elderly. Abnormality in LDL, HDL, and Cr levels might contribute to the depressive symptoms.

## INTRODUCTION

Depression, ranging widely from mild depressive symptoms to clinically diagnosed major depression, mainly characterized by depressed mood and loss of willpower, and accompanied by significant somatic or cognitive impairment, is an increasingly serious global problem [1, 2]. Approximately 20%–51% of patients with coronary heart disease (CHD) have been afflicted by clinical depression or depressive symptoms, which are significantly higher than that of 4.3% of the local population estimated by the World Health Organization (WHO, 2017) [3, 4]. However, the causal linkage between CHD and depression is still incompletely understood [5]. CHD accompanied by depression may be caused by multiple factors, including physiological and psychosocial factors, such as inflammation, endothelial dysfunction, hypothalamic–pituitary–adrenal axis hyperactivity, various behavioral factors, gut microbiome, and endocrine signaling [5, 6].

In patients with stable coronary artery disease (SCAD), the quality of life and health conditions decline sharply when complicated by depression, and depression is also one of the independent risk factors for adverse cardiovascular events [5, 7, 8]. Indeed, the 2012 American Heart Association Guidelines and 2013 European Society of Cardiology Guidelines on the management of SCAD (also known as stable ischemic heart disease) have already emphasized the need to screen depression in patients with SCAD and the necessity to provide appropriate interventions [9, 10]. However, because of the lack of necessary and sufficient understanding, depressive symptoms are usually masked under physical illness or manifest as severe somatic symptoms inconsistent with disease severity, for which making a correct diagnosis and taking appropriate intervention are difficult for clinicians.

To date, there are no definite data on the prevalence of depressive symptoms in patients with SCAD, particularly in the Chinese population. We investigated the prevalence rate and severity of depressive symptoms in patients with SCAD and identified the risk factors related to depressive symptoms by comparing clinical characteristics among patients to clarify the relationship between SCAD and depressive symptoms and to increase the attention of clinicians to the comorbidity.

## RESULTS

### Clinical characteristics of patients with SCAD in the non-depressive and depressive groups

**Figure 1:**
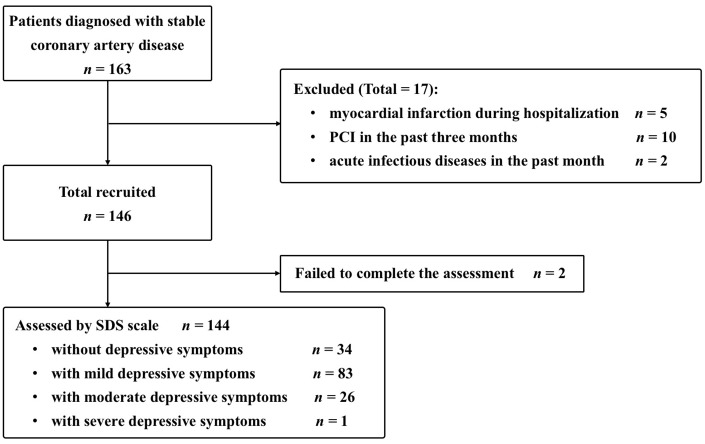
**Inclusive cases. PCI, percutaneous coronary intervention; SDS, Self-Rating Depression Scale.**

Of 163 patients with SCAD at baseline, 19 were excluded from this analysis: 5 with myocardial infarction during hospitalization, 10 with percutaneous coronary intervention (PCI) in the past 3 months, 2 with acute infectious diseases in the past month, and 2 who failed to complete the Zung Self-Rating Depression Scale (SDS) (Figure 1). No significant differences were found in the demographic and clinical characteristics between individuals and those excluded. The study sample was composed of 144 patients with SCAD, and the rates of no depressive symptoms, mild depressive symptoms, and moderate/severe depressive symptoms were 23.6%, 57.6%, and 18.8%, respectively.

Table 1 shows the clinical characteristics of the two groups. The mean age of patients in the depressive group was older than that of those in the non-depressive group (*P* = 0.007). No significant differences were found between the two groups in other parameters. However, higher incidence of diabetes mellitus and higher Gensini scores were noted in the depressive group than those in the non-depressive group, although the differences did not show a statistical significance.

**Table 1:** Clinical characteristics of SCAD patients with or without depressive symptoms.

**Parameters**	**non-depressive symptoms group (n = 34)**	**depressive symptoms group (n = 110)**	***P* value**
Age (years)	62.2 ± 14.2	63.2 ± 8.6	0.007
Male (%)	23(67.6)	79(71.8)	0.640
Systolic blood pressure (mmHg)	136 ± 19	134 ± 18	0.982
Diastolic blood pressure (mmHg)	80 ± 13	79 ± 10	0.236
BMI (kg/m^2^)	25.0 ± 3.2	24.6 ± 3.1	0.961
Diabetes mellitus (%)	8(23.5)	38(34.5)	0.229
Current smoking (%)	10(29.4)	40(36.4)	0.457
ALT (U/L)	27.5 ± 16.4	24.1 ± 14.7	0.195
AST (U/L)	25.1 ± 8.5	23.0 ± 7.6	0.151
STB (μm/L)	10.6 ± 3.5	10.4 ± 4.9	0.358
High density lipoprotein (mmol/L)	1.01 ± 0.22	2.13 ± 1.25	0.329
Low density lipoprotein (mmol/L)	2.12 ± 0.76	1.97 ± 0.64	0.300
A/G	1.69 ± 0.37	2.00 ± 0.37	0.495
BUN (mg/dl)	6.72 ± 2.77	7.39 ± 2.43	0.787
Cr (μmmol/L)	80.8 ± 30.0	75.5 ± 45.6	0.081
Hb (g/L)	138.1 ± 12.7	139.3 ± 12.0	0.809
WBC (×10^9^/L)	6.82 ± 1.73	6.41 ± 1.49	0.206
N (%)	61.6 ± 7.2	64.6 ± 8.6	0.297
L (%)	28.8 ± 9.5	26.0 ± 8.7	0.626
BNP (pg/ml)	76.5 ± 57.1	63.4 ± 54.8	0.856
Medications (%)			
β Blockers	9(26.5)	44(40.0)	0.153
Statins	15(44.1)	52(47.3)	0.747
LVEF (%)	61.7 ± 3.0	61.6 ± 2.77	0.841
Gensini scores	22.10 ± 13.99	28.69 ± 22.28	0.370

A/G, albumin/globulins; ALT, alanine aminotransferase; AST, aspartate aminotransferase; BMI, body mass index; BNP, brain natriuretic peptide; BUN, blood urea nitrogen; Cr, creatinine; Hb, hemoglobin; L%, lymphocyte percentage; LVEF, left ventricular ejection fraction; N%, neutrophil percentage; SCAD, stable coronary artery disease; STB, blood total bilirubin; WBC, white blood cell.

### Clinical characteristics of patients with SCAD in the non-depressive and mild depressive groups

Table 2 shows the clinical characteristics of the two groups. The mean age of patients in the mild depressive group was older than that of those in the non-depressive group (*P* = 0.012). No significant differences were found between the two groups in other parameters. However, higher incidence of diabetes mellitus and higher Gensini scores were noted in the mild depressive group than those in the non-depressive group, although the differences did not show a statistical significance.

**Table 2:** Clinical characteristics of SCAD patients without or with mild depressive symptoms.

**Parameters**	**non-depressive symptoms group (n = 34)**	**mild depressive symptoms group (n = 83)**	***P* value**
Age (years)	62.2 ± 14.2	63.3 ± 8.5	0.012
Male (%)	23(67.6)	61(73.5)	0.523
Systolic blood pressure (mmHg)	136 ± 19	132 ± 18	0.747
Diastolic blood pressure (mmHg)	80 ± 13	78 ± 10	0.121
BMI (kg/m^2^)	25.0 ± 3.2	24.6 ± 3.2	0.912
Diabetes mellitus (%)	8(23.5)	29(34.9)	0.228
Current smoking (%)	10(29.4)	32(38.6)	0.349
ALT (U/L)	27.5 ± 16.4	22.6 ± 12.9	0.080
AST (U/L)	25.1 ± 8.5	22.1 ± 7.2	0.051
STB (μm/L)	10.6 ± 3.5	10.6 ± 4.8	0.544
High density lipoprotein (mmol/L)	1.01 ± 0.22	1.03 ± 0.25	0.792
Low density lipoprotein (mmol/L)	2.12 ± 0.76	1.94 ± 0.64	0.259
A/G	1.69 ± 0.37	1.81 ± 0.67	0.538
BUN (mg/dl)	4.95 ± 1.65	5.07 ± 1.75	0.782
Cr (μmmol/L)	80.8 ± 30.0	81.3 ± 48.8	0.303
Hb (g/L)	138.1 ± 12.7	139.0 ± 12.5	0.928
WBC (×10^9^/L)	6.82 ± 1.73	6.48 ± 1.50	0.315
N (%)	61.6 ± 7.2	65.1 ± 8.4	0.502
L (%)	28.8 ± 9.5	25.3 ± 8.6	0.515
BNP (pg/ml)	76.5 ± 57.1	65.5 ± 55.2	0.572
Medications (%)			
β Blockers	9(26.5)	33(39.8)	0.174
Statins	15(44.1)	35(42.2)	0.847
LVEF (%)	61.7 ± 3.0	61.8 ± 4.1	0.861
Gensini scores	22.10 ± 13.99	27.33 ± 20.85	0.593

A/G, albumin/globulins; ALT, alanine aminotransferase; AST, aspartate aminotransferase; BMI, body mass index; BNP, brain natriuretic peptide; BUN, blood urea nitrogen; Cr, creatinine; Hb, hemoglobin; L%, lymphocyte percentage; LVEF, left ventricular ejection fraction; N%, neutrophil percentage; SCAD, stable coronary artery disease; STB, blood total bilirubin; WBC, white blood cell.

Table 3 shows the risk factors associated with mild depressive symptoms among patients with SCAD via binary logistic regression analysis. Lower low-density lipoprotein (LDL) levels (odds ratio [OR], 0.338; 95% confidence interval [CI], 0.131–0.875; *P* = 0.025), lower white blood cell (WBC) count (OR, 0.617; 95% CI, 0.430–0.884; *P* = 0.009), and β-blocker medication (OR, 0.236; 95% CI, 0.063–0.885; *P* = 0.032) were significantly associated with increased risks of mild depressive symptoms.

**Table 3:** Association of mild depressive symptoms and clinical characteristics.

**Parameters**	**B**	**OR (95% CI)**	***P* value**
Age (years)	-0.012	0.988 (0.938–1.041)	0.653
Male (%)	−0.558	0.572 (0.125–2.627)	0.473
Systolic blood pressure (mmHg)	0.006	1.006 (0.966–1.049)	0.758
Diastolic blood pressure (mmHg)	−0.035	0.966 (0.904–1.032)	0.303
BMI (kg/m^2^)	0.002	1.002 (0.841–1.193)	0.982
Diabetes mellitus (%)	−0.0497	0.608 (0.191–1.943)	0.402
Current smoking (%)	−0.959	0.383 (0.106–1.384)	0.143
ALT (U/L)	0.018	1.019 (0.967–1.073)	0.485
AST (U/L)	−0.114	0.892 (0.797–0.998)	0.051
STB (μm/L)	−0.016	0.984 (0.864–1.121)	0.811
High density lipoprotein (mmol/L)	1.192	4.914 (0.439–54.992)	0.196
Low density lipoprotein (mmol/L)	−1.084	0.338 (0.131–0.875)	0.025
A/G	0.018	1.018 (0.716–1.448)	0.919
BUN (mg/dl)	−0.082	0.921 (0.671–1.265)	0.613
Cr (μmmol/L)	0.003	1.003 (0.991–1.016)	0.576
Hb (g/L)	0.034	1.035 (0.985–1.087)	0.172
WBC (×10^9^/L)	−0.483	0.617 (0.430–0.884)	0.009
N (%)	0.085	1.089 (0.968–1.225)	0.157
L (%)	0.012	1.012 (0.917–1.117)	0.815
BNP (pg/ml)	−0.004	0.996 (0.989–1.004)	0.345
Medications (%)			
β Blockers	−1.443	0.236 (0.063–0.885)	0.032
Statins	1.204	3.335 (0.861–12.917)	0.081
LVEF (%)	−0.036	0.964 (0.822–1.131)	0.654
Gensini scores	0.016	1.016 (0.992–1.041)	0.186

A/G, albumin/globulins; ALT, alanine aminotransferase; AST, aspartate aminotransferase; BMI, body mass index; BNP, brain natriuretic peptide; BUN, blood urea nitrogen; Cr, creatinine; Hb, hemoglobin; L%, lymphocyte percentage; LVEF, left ventricular ejection fraction; N%, neutrophil percentage; SCAD, stable coronary artery disease; STB, blood total bilirubin; WBC, white blood cell.

### Clinical characteristics of patients with SCAD in the non-depressive and moderate/severe depressive groups

Table 4 shows the clinical characteristics of the two groups. Patients with SCAD with moderate/severe depressive symptoms had higher HDL levels (*P* = 0.022) and lower creatinine (Cr) levels (*P* = 0.027). No significant differences were found between the two groups in other parameters. However, higher incidence of diabetes mellitus and higher Gensini scores were noted in the moderate/severe depressive group than those in the non-depressive group, although the differences did not show a statistical significance.

**Table 4:** Clinical characteristics of SCAD patients without or with moderate/severe depressive symptoms.

**Parameters**	**non-depressive symptoms group (n = 34)**	**moderate/severe depressive symptoms group (n = 27)**	***P* value**
Age (years)	62.2 ± 14.2	62.8 ± 9.0	0.099
Male (%)	23(67.6)	18(66.7)	0.935
Systolic blood pressure (mmHg)	136 ± 19	140 ± 19	0.776
Diastolic blood pressure (mmHg)	80 ± 13	83 ± 11	0.855
BMI (kg/m^2^)	25.0 ± 3.2	24.7 ± 2.9	0.895
Diabetes mellitus (%)	8(23.5)	9(33.3)	0.396
Current smoking (%)	10(29.4)	8(29.6)	0.985
ALT (U/L)	27.5 ± 16.4	28.7 ± 18.7	0.816
AST (U/L)	25.1 ± 8.5	25.7 ± 8.4	0.765
STB (μm/L)	10.6 ± 3.5	9.8 ± 5.2	0.168
High density lipoprotein (mmol/L)	1.01 ± 0.22	1.14 ± 0.24	0.022
Low density lipoprotein (mmol/L)	2.12 ± 0.76	2.04 ± 0.66	0.665
A/G	1.69 ± 0.37	1.62 ± 0.25	0.647
BUN (mg/dl)	4.95 ± 1.65	5.30 ± 2.75	0.902
Cr (μmmol/L)	80.8 ± 30.0	68.7 ± 13.0	0.027
Hb (g/L)	138.1 ± 12.7	140.3 ± 10.4	0.316
WBC (×10^9^/L)	6.82 ± 1.73	6.21 ± 1.46	0.127
N (%)	61.6 ± 7.2	63.0 ± 9.3	0.168
L (%)	28.8 ± 9.5	28.0 ± 8.8	0.851
BNP (pg/ml)	76.5 ± 57.1	56.9 ± 54.2	0.420
Medications (%)			
β Blockers	9(26.5)	11(40.7)	0.238
Statins	15(44.1)	17(62.9)	0.143
LVEF (%)	61.7 ± 3.0	61.0 ± 7.3	0.843
Gensini scores	22.10 ± 13.99	32.87 ± 26.20	0.140

A/G, albumin/globulins; ALT, alanine aminotransferase; AST, aspartate aminotransferase; BMI, body mass index; BNP, brain natriuretic peptide; BUN, blood urea nitrogen; Cr, creatinine; Hb, hemoglobin; L%, lymphocyte percentage; LVEF, left ventricular ejection fraction; N%, neutrophil percentage; STB, blood total bilirubin; WBC, white blood cell.

Table 5 shows the risk factors associated with moderate/severe depressive symptoms among patients with SCAD via binary logistic regression analysis. Higher high-density lipoprotein (HDL) levels (OR, 82.553; 95% CI, 1.311–5196.951; *P* = 0.037) and lower Cr levels (OR, 0.876; 95% CI, 0.774–0.986; *P* = 0.029) were significantly associated with increased risks of moderate/severe depressive symptoms.

**Table 5:** Association of moderate/severe depressive symptoms and clinical characteristics.

**Parameters**	**B**	**OR (95% CI)**	***P* value**
Age (years)	0.104	1.110 (0.923–1.334)	0.269
Male (%)	0.194	1.214 (0.130–11.358)	0.865
Systolic blood pressure (mmHg)	−0.044	0.957 (0.882–1.040)	0.301
Diastolic blood pressure (mmHg)	0.044	1.045 (0.925–1.182)	0.479
BMI (kg/m^2^)	−0.218	0.804 (0.581–1.112)	0.187
Diabetes mellitus (%)	−0.228	0.796 (0.107–5.935)	0.824
Current smoking (%)	−0.769	0.464 (0.44–4.928)	0.524
ALT (U/L)	0.114	1.121 (0.979–1.284)	0.098
AST (U/L)	0.214	0.807 (0.629–1.036)	0.093
STB (μm/L)	−0.053	0.949 (0.768–1.171)	0.623
High density lipoprotein (mmol/L)	4.413	82.553 (1.311–5196.951)	0.037
Low density lipoprotein (mmol/L)	−0.130	0.878 (0.225–3.433)	0.852
A/G	0.206	1.229 (0.037–40.618)	0.908
BUN (mg/dl)	0.002	1.002 (0.528–1.903)	0.994
Cr (μmmol/L)	−0.135	0.874 (0.774–0.986)	0.029
Hb (g/L)	0.099	1.104 (0.978–1.245)	0.109
WBC (×10^9^/L)	−0.481	0.618 (0.342–1.118)	0.111
N (%)	0.054	1.056 (0.828–1.346)	0.661
L (%)	0.058	1.060 (0.865–1.298)	0.573
BNP (pg/ml)	−0.013	0.987 (0.973–1.002)	0.099
Medications (%)			
β Blockers	−0.329	0.720 (0.048–10.724)	0.811
Statins	−2.173	0.114 (0.009–1.524)	0.101
LVEF (%)	−0.245	0.783 (0.567–1.081)	0.137
Gensini scores	0.028	1.028 (0.999–1.059)	0.063

A/G, albumin/globulins; ALT, alanine aminotransferase; AST, aspartate aminotransferase; BMI, body mass index; BNP, brain natriuretic peptide; BUN, blood urea nitrogen; Cr, creatinine; Hb, hemoglobin; L%, lymphocyte percentage; LVEF, left ventricular ejection fraction; N%, neutrophil percentage; STB, blood total bilirubin; WBC, white blood cell.

## DISCUSSION

The results of this study showed that the incidence of depressive symptoms in patients with SCAD were significantly higher than that in the local population, which was estimated by the WHO (2017), and the rate of moderate/severe depressive symptoms was 18.8%. Interestingly, elderly patients were prone to experience depressive symptoms, and abnormality in LDL, HDL, and Cr levels might contribute to the depressive symptoms. Therefore, elderly patients with SCAD with LDL, HDL, and Cr abnormalities would promote the occurrence of depressive symptoms.

An increasing number of studies have suggested that age was an independent risk factor for the development of mood disorders, especially depression [11, 12], which may be highly related to the emotional instability caused by thalamic dysfunction in the elderly [13]. A 2 year follow-up study by Schaakxs et al. reported that older patients had a worse course of depressive disorder than younger patients, which was mainly manifested in the presence of any depression diagnosis (OR, 1.08; 95% CI, 1.00–1.17), chronic symptom course (OR, 1.24; 95% CI, 1.13–1.35), time to remission (hazard ratio, 0.91; *P <* 0.0001), and depression severity change (regression coefficient, 1.06; *P <* 0.0001), all of which were still mostly significant after adjusting for prognostic clinical, social, and health factors [12]. Furthermore, elderly people are more likely to take antidepressant drugs rather than psychotherapy when experiencing depression [14]. However, antidepressants could lead to dyslipidemia, thereby exacerbating cardiovascular diseases [15–17]. Moreover, the interaction between drugs and various chronic diseases (such as metabolic syndrome and cardiovascular disease) in elderly patients would have an influential impact on the effectiveness and risk of antidepressants [18]. These factors may affect the outcome of patients with SCAD accompanied by depressive symptoms. In addition, a known factor affecting the pathogenesis of depression in the elderly is cognitive decline, through multiple mechanisms, including hippocampal abnormalities, white matter hyperintensities, and amyloid beta levels [19, 20]. Therefore, assessment of cognitive decline is critical in subsequent studies attempting to reveal the role of age in the development of depressive symptoms in patients with SCAD.

Lipoprotein plays an important role in the development of CHD. Cholesterol in atherosclerotic plaque was proven to be derived from serum LDL, deemed as the atherogenic lipid. By contrast, HDL was considered to be the anti-atherosclerotic lipid, acting as a protective factor for CHD [21, 22]. Interestingly, the results of this study indicate that lower LDL and higher HDL levels in patients with SCAD are risk factors for depressive symptoms. Previous studies based on the data from the Korean National Health and Nutrition Examination Survey have identified that adjusted for other covariates, lower LDL and higher HDL levels were significantly associated with increased risk of depressive symptoms [23, 24]. Similarly, Persons et al. showed that lowering LDL levels could increase risk of depression, and improving the psychological status of patients could help regulate blood lipid levels [25]. The above evidence is consistent with the findings in the present study. Furthermore, after adjustment of demographic variables and cardiovascular risk factors, depression was associated with an increase in calcified plaque by measurement using coronary computed tomography angiography [26], which fully displayed the relationship between depression and atherosclerosis. The linkage between depressive symptoms and LDL and HDL levels may be due to differences in serotonin function and activity. Troisi et al. reported that cholesterol not only was a major component of cell membrane and myelin but also played a vital role in the development, activity, and stability of synapses [27]. Reduced cholesterol levels in the brain cell membrane would cause a decrease in lipid viscosity, which affects the exposure of serotonin receptors, thereby leading to depression [27]. Given that LDL and HDL levels are risk factors for depressive symptoms in patients with SCAD, increasing attention should be paid to the pathological basis of atherosclerosis and the risk of depression, as well as considering possible effects of lipid-lowering drugs on both aspects.

In this study, decreased Cr levels are risk factors for the onset of moderate/severe depressive symptoms, which are consistent with previous findings [28], which might be related to anorexia nervosa. Serum Cr has been known to be produced from both exogenous and endogenous processes. Exogenous Cr is the product of the digestible meat, whereas endogenous Cr is formed through irreversible non-enzymatic dehydration during muscle metabolism. Cr levels are relatively stable when there is a balance between meat intake and muscle metabolism but without renal failure (one of the exclusion criteria for this study) [29, 30]. Depression could lead to loss of appetite, reduced food intake, and decreased digestion function, which causes lower exogenous Cr levels. In addition, low mood and loss of willpower cause patients with depression to avoid social events and active exercise, thereby slowing muscle metabolism activity, which finally causes lower endogenous Cr levels [31, 32]. In this regard, clinical doctors must encourage patients to increase food intake and participate in exercise actively to increase the serum Cr levels, thereby improving depressive symptoms.

Furthermore, although the differences did not show a statistical significance, higher incidence of diabetes mellitus and higher Gensini scores were noted in the depressive group (both mild and moderate/severe groups), indicating that diabetes mellitus might be related to the occurrence of SCAD accompanied by depressive symptoms, and depressive symptoms could aggravate the degree of coronary artery stenosis. Given that these are all involved in inflammation, metabolism disorder, endothelial dysfunction, and platelet activation [5, 6, 33], a complex pathogenic network might likely exist in SCAD, diabetes mellitus, and depressive symptoms. Moreover, after searching the literature from January 1946 to December 2014, Doi-Kanno et al. showed that diabetes mellitus was a possible risk factor for depression in patients with myocardial infarction who underwent PCI [34]. In addition, numerous systematic reviews and prospective analyses agree that the involvement of depression could translate diabetes into increased CHD risk [33]. Thus, in future studies, more attention should be paid on patients with comorbid diseases, and appropriate interventions should be performed on patients with SCAD with predisposing factors playing a significant role in the progress of depressive symptoms.

The present study has some limitations. First, we did not assess other mental comorbidities and depressive symptoms by using other scales. Although SDS is one of the most widely used self-report scale, and its validity have been established in clinical depression evaluation [35–37], our results would have been more reliable if combined with the use of other scales. Second, we did not obtain data on some variables, such as social support, economic status, or marital status, which may influence the result to a certain extent. Third, it was a single-center, small-size study, and the patients recruited were from one hospital only, thereby raising concerns on generality from its findings. In addition, the significance of *P* value is discounted because of the strong standard deviations (SDs) in statistical analysis, which affects the reliability of the conclusions. Fourth, as a cross-sectional study, the results cannot affirm a causal relationship between depressive symptoms and the above-mentioned biochemical parameters. Future cohort studies will be necessary.

In conclusion, depressive symptoms are common among patients with SCAD, particularly in the elderly. Abnormalities in LDL, HDL, and Cr levels were significantly associated with the occurrence of depressive symptoms. These results underscore the importance of screening depressive symptoms in patients with SCAD and propose the potential intervention mechanisms of preventing the comorbidity. Large-scale prospective interventional studies should be encouraged to determine whether regulating LDL, HDL, and Cr levels can actually improve depressive symptoms of the SCAD population.

## MATERIALS AND METHODS

### Setting and participants

The study was a cross-sectional, observational, and single-center study. The patients surveyed in this study were randomly selected from all patients with SCAD in the Third Affiliated Hospital of Soochow University between July 2017 and October 2018. The study was approved by the Ethics Committee of the Third Affiliated Hospital of Soochow University and has been registered in the Chinese Clinical Trial Registry (ChiCTR1900020594).

Patients with SCAD meeting at least one of the following criteria were enrolled: (1) clinically diagnosed with myocardial infarction (>3 months); (2) coronary angiography showing a stenosis of >50% in one or more coronary arteries; (3) typical chest pain and noninvasive examination provided evidence of coronary artery stenosis or myocardial ischemia; and (4) underwent coronary artery bypass grafting or PCI (>3 months). All patients should have no evidence of myocardial injury (elevated myocardial enzymes) at the moment. The patients were able to complete the depression scale assessment and signed informed consent.

Exclusion criteria were as follows: (1) severe liver or kidney failure; (2) history of depression or other psychiatric disorders (on anti-depressant medication, clinical diagnosis, or previous treatment); (3) acute infectious disease within the past month; (4) other severe cardiovascular diseases, such as acute pericarditis, myocarditis, end-stage heart failure, or secondary heart disease; (5) diseases seriously affecting life expectancy (such as connective tissue disease, tumors, drug abuse, and dementia); (6) pregnant women; and (7) recent major stressful life events, trauma, or surgery.

### Physical and clinical examination

Baseline data were obtained through interviews, medical records, and actual measurement, including age, sex, body mass index, blood pressure, diabetes history, smoking history, and β blocker and statin medication. Fasting venous blood samples were collected and sent to our laboratory for measurement of alanine aminotransferase, aspartate aminotransferase, blood total bilirubin, HDL, LDL, albumin/globulins, blood urea nitrogen, Cr, hemoglobin, WBC, neutrophil percentage, lymphocyte percentage, and brain natriuretic peptide. All patients underwent echocardiography to obtain left ventricular ejection fraction, and all the tests were performed and reported by the same physician in the hospital. All patients underwent coronary angiography via the brachial or radial artery, and the results were determined by two experienced cardiologists. The degree of stenosis of the left main artery, left anterior descending artery, circumflex artery, and right coronary artery lumen was recorded, and the Gensini score was calculated to quantitatively evaluate the degree of coronary artery stenosis.

### Assessment of depression symptoms

Patients with SCAD were evaluated for depressive symptoms by using SDS during hospitalization. SDS is a self-reported clinical scale consisting of 20 items and scored on a 4-point scale to assess the psychological and physical symptoms of depression. The patients were categorized into three subgroups based on the results of SDS: non-depressive (score ≤52), mild depressive (score ≥53 but ≤62), and moderate/severe depressive groups (score ≥63).

### Statistical analysis

Statistical analyses were performed using SPSS 24.0. Continuous data were presented as mean ± SD and compared using Student t test or Mann–Whitney U test. The categorical data were presented as percentage frequency (%) and compared using chi-squared test. Binary logistic regression analyses were used to determine the factors associated with depressive symptoms. The results were presented as ORs and 95% CIs. A two-tailed *P* < 0.05 indicates statistical significance.
